# Creating a Metabolic Syndrome Research Resource using the National Health and Nutrition Examination Survey

**DOI:** 10.1093/database/baaa103

**Published:** 2020-12-31

**Authors:** Willysha S Jenkins, Christian Richardson, Ariel Williams, Clarlynda R Williams-DeVane

**Affiliations:** Department of Mathematics and Computer Science Fisk University, Nashville, TN 37208, USA; Duke School of Medicine Duke University, Durham, NC 27708, USA; Department of Integrated Biosciences North Carolina Central University, Durham, NC 27707, USA; Department of Mathematics and Computer Science Fisk University, Nashville, TN 37208, USA; Duke School of Medicine Duke University, Durham, NC 27708, USA

## Abstract

Metabolic syndrome (MetS) is multifaceted. Risk factors include visceral adiposity, dyslipidemia, hyperglycemia, hypertension and environmental stimuli. MetS leads to an increased risk of cardiovascular disease, type 2 diabetes and stroke. Comparative studies, however, have identified heterogeneity in the pathology of MetS across groups though the etiology of these differences has yet to be elucidated. The Metabolic Syndrome Research Resource (MetSRR) described in this report is a curated database that provides access to MetS-associated biological and ancillary data and pools current and potential biomarkers of MetS extracted from relevant National Health and Nutrition Examination Survey (NHANES) data from 1999–2016. Each potential biomarker was selected following the review of over 100 peer-reviewed articles. MetSRR includes 28 demographics, survey and known MetS-related variables, including 9 curated categorical variables and 42 potentially novel biomarkers. All measures are captured from over 90 000 individuals. This biocuration effort provides increased access to curated MetS-related data and will serve as a hypothesis-generating tool to aid in novel biomarker discovery. In addition, MetSRR provides the ability to generate and export ethnic group-/race-, sex- and age-specific curated datasets, thus broadening participation in research efforts to identify clinically evaluative MetS biomarkers for disparate populations. Although there are other databases, such as BioM2MetDisease, designed to explore metabolic diseases through analysis of miRNAs and disease phenotypes, MetSRR is the only MetS-specific database designed to explore etiology of MetS across groups, through the biocuration of demographic, biological samples and biometric data.

**Database URL:**  http://www.healthdisparityinformatics.com/MetSRR

## Introduction

Health disparities have been defined as ‘differences in the incidence, prevalence, mortality, burden of diseases as well as other adverse health conditions that exist among specific population groups…’ (1). According to the American Hospital Association, the health disparity conundrum continues to force scientists and clinicians to push the bounds of knowledge discovery, clinical observation and application. Common populations linked to health disparities include self-identified racial or ethnic groups, gender, sexual orientation, age, disability status, socioeconomic status and geographic location (2). Patterns of variation among these populations have been linked to an array of complex diseases and syndromes such as Metabolic Syndrome (MetS).

Common types of health disparities associated with MetS include age and socioeconomic status (3). In a recent study addressing correlations between objective socioeconomic status (OSS) and MetS, it was found that OSS was independently associated with MetS (4). In addition to OSS, studies have shown that the incidence of MetS is higher in overweight and obese minority populations (5). Differences across biological sex and self-identified race have been noted but concrete causality among these variables and the incidence of MetS has yet to be established. Due to racial differences and the lack of causative identifiers, it is imperative to identify clinically relevant evaluative MetS biomarkers (6). New and innovative approaches, such as informatics, can help reveal clinically relevant MetS biomarkers, integrating molecular data via hybrid methodologies to useful diagnostic mosaics of MetS biomarkers for clinical diagnostic and prognostic value.

Informatics is the science of processing data for storage and retrieval. It applies principles from computer science and information science for the advancement of research in areas of life sciences. The aim was to provide access to large amounts of data that can be computationally evaluated to reveal patterns, trends and associations. Domain-specific areas of informatics such as biomedical informatics focus on developing and implementing technologies that assist researchers and clinicians with data relevant to specific research and disease areas. Health-care research programs, academic research departments and governmental agencies have successfully amassed large amounts of health-related data across various demographic communities for these purposes of addressing health disparities and disease. Databases such as the National Health Interview Survey (NHIS), Medical Expenditure Panel Survey (MEPS), Behavioral Risk Factor Surveillance System (BRFSS) and National Health and Nutrition Examination Survey (NHANES) house copious amounts of health-related demographic, biological sample, clinical biometric (3) and other ancillary data for exploration. However, despite the presence of these resources, it is still rare to find clinicians, basic science researchers and other scholars utilizing human data to advance our knowledge in the etiology of MetS and subsequent disease(s).

One noted challenge in advancing our knowledge of MetS is accessibility to quality human data. The process of querying databases for disease-specific genes or biometric variables can be multifaceted and complex. Wrangling and cleaning data are processes that can often be cumbersome and time-consuming, especially when faced with data that were collected by others. When surveyed about accessing and analyzing human data found via databases, even the most computationally savvy researchers expressed that wrestling with software and wrangling big data into workable formats prior to analysis were both stressful and time-consuming (7). There is a great need to improve this process and increase the accessibility to MetS biomarkers by curating data to eliminate these challenges of data access and cleaning and provide it in a central location that links the curated data to a comprehensive review of the literature.

Recent literature indicates that techniques used in biomedical informatics should be employed as rapid identification tools for classifying MetS (8). Currently, there are databases, such as BioM2MetDisease, that explore associations with various metabolic diseases with biomolecules. However, these databases do not explore associations of the biomolecules with MetS, the precursor to metabolic diseases. Therefore, we present the alpha phase of the open-source Metabolic Syndrome Research Resource (MetSRR). MetSRR is a curated database that provides access to MetS-associated biological and ancillary data. It is an amalgamation of current and potential biomarkers of MetS extracted from relevant NHANES data from 1999–2016. Each potential biomarker was selected following the review of over 100 peer-reviewed articles. MetSRR includes 28 demographics, survey and known MetS-related variables, including 9 curated categorical variables and 42 potentially novel biomarkers. All measures are captured from over 90 000 individuals.

Our database has two components, the MetSRR Explorer and the MetSRR Tables. MetSRR Explorer gives users the ability to curate, download and analyze customized datasets pertinent to their research or clinical interests. Features of the Explorer also include data visualization, providing at-a-glance insight to the distribution of variables and basic descriptive statistics. MetSRR Tables provide a full characterization of each variable, including an evaluation of missing values, descriptive statistics and access to literature explanative of its inclusion.

Our MetS-specific database functions as an information and resource hub that contributes to the assessment of disparities in MetS. Our goal was to provide a resource that elucidates the etiological variability in MetS. It is common practice for researchers to ascribe the task of experimental design and data analysis to data analysts, biostatisticians and informaticians. Efficient experimental design and accurate data analysis can be inefficient when isolated. The process of translating and integrating biological information into user-friendly databases and resources has become and will remain an essential part of biological discovery and biomedical research. For complex diseases and syndromes such as MetS, curation allows for researchers of varying expertise to participate in the extraction of knowledge from otherwise unstructured data.

## Materials and methods

### Curation procedure

#### Identification of publicly available data

Identifying resources from which MetSRR datasets could be curated required that the resource: (i) had open-source publicly available data, (ii) had protocols that allowed for the pooling of variables including demographic surveys, socioeconomic status surveys, basic health information surveys and various clinical measurements and (iii) most importantly had biometric variables. Existing resources include the NHIS, MEPS, BRFSS and NHANES (9). A review of the literature allowed us to determine which resource was optimal for our biocuration efforts. NHIS was eliminated because the data collected were not laboratory intensive. Data collection methods for NHIS are strictly focused on health expenditure data and thus lacked a number of objective/quantifiable data. The MEPS resource was excluded because it primarily collects data related to health surveillance, namely costs, health payment methods, insurance and health service utilization, and thus does not offer data assets relevant to the biocuration focus of this database. Lastly, BRFSS was eliminated because its methods focused on the collection of health risk-related data. Finally, NHANES was evaluated and selected as a resource for our efforts as it included all key elements listed in the four criteria above. NHANES utilizes an established, streamlined interview process that is inclusive of minority groups across the country. Individuals were given numeric identifiers allowing follow-up measurements to be taken over time. Most importantly, the data collected include demographic surveys, socioeconomic status surveys, basic health information surveys, various clinical measurements and biometric variables (9).

#### Identification of primary programming language

The criteria for selecting a programming language for creating MetSRR includes the following characteristics: the language must be easy to install, be user-friendly, have the capacity to integrate other programming languages, include easily accessible statistical packages, allow manipulation of large datasets and be easy to update. The selected programming language must also have enhanced sampling and visualization capabilities, features that contribute to the reproducibility of curation by new users. Finally, the language must be open source.

There were three common statistical software programs considered in this process: R, the Statistical Analysis System (SAS) and the Statistical Package for the Social Sciences (SPSS). Upon evaluation, both SAS and SPSS were excluded as options. Cost proved to be a major factor when considering SAS with commercial use having a first-year fee upwards of $4000. There are also additional fees for using various statistical algorithms and text-mining software. Reviews of SPSS allude to a steep learning curve for novices. Unlike R, SPSS does not have a large community that provides free online sources. SPSS also lacks the ability to handle large datasets. Neither SAS nor SPSS integrates easily with other languages and can be challenging or costly to update.

Ultimately, we selected R as the programming language for creating MetSRR. R is available to users at no charge (10). It is a resource that is increasingly being used by statisticians and informaticians for developing statistical programs and conducting data analysis (11). Currently R is ranked 9th in the TIOBE Index, a measure of the popularity of programming languages (12). R has a command-line interface with multiple graphic user interfaces (GUIs) on top, commonly referred to as R Studio. This integrated development environment allows for ease of use and (13) reproducibility for data explorers.

## MetSRR creation

### Variable selection procedure

#### Cross-referencing of annual data files

**Figure 1.**
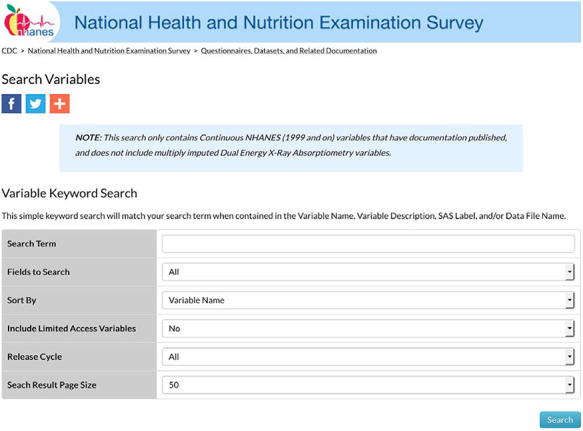
NHANES variable search landing page.

The first step in the creation of MetSRR was cross-referencing variables. Each variable considered for inclusion was verified for its presence in the NHANES database for each cycle. An NHANES cycle spans a 2-year period. As stated above, MetSRR includes years 1999 through 2016. The cycles were defined as: cycle 1 covers years 1999 through 2000, cycle 2 covers 2001–2002, cycles 3 covers 2003–2004 and so on until 2016 is reached. Over the years, variable names and sample collection methods changed. Therefore, variables were cross-referenced across cycles. As of January 2019, NHANES made this process more streamlined by updating the variable names and search options. Variable search option was assessed by the following link: https://wwwn.cdc.gov/nchs/nhanes/Default.aspx. The variable search link routes to a platform with additional filters such as: Search Term, Fields to Search, Sort By, Include Limited Access Variables, Release Cycle and Search Result Page Size options. Figure 1 depicts the landing page.

**Figure 2.**
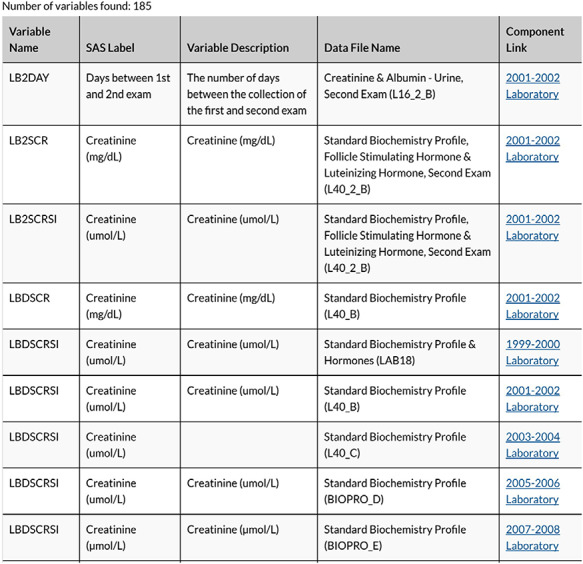
Snapshot of variable search results for creatinine.

Each variable considered for inclusion was entered into the Search Term field. The designation for all cycle years (All) was selected for the Release Cycle field so as to access all available cycles’ measurements associated with the variable name. Figure 2 illustrates a sample output, a Table with every file with the variable included will populate. In Figure 2, the Variable Name column provides the variable code name assigned by NHANES. Also, in that Table NHANES provides the Data File Name, SAS Label, Variable Description and a Component Link.

We selected the component link items for each variable to allow for more information about data sample collection procedures and additional variables to be included in the data file. The component link led to a Table with the binary .xpt files available for download.

There are various descriptive abbreviations for data files within MetSRR. Data files names are only differentiated by the last letter of the file name, which designates the cycle. Variable code names were often the same; however, we adjusted if collection procedures or sample processing methods changed. An example of changes in identification of a particular variable was seen with MetSRR’s HDL variable. From 99-02 NHANES identified this variable as LBDHDL. However, in 03-04, the HDL variable changed to LBXHDD and has since been coded as LBDHDD. All NHANES code names for the variables and differences were recorded; similarly, during the cross-referencing phase, all variables represented four or more cycles were included.

#### Selection of sample type and unit of measure

It is NHANES’ practice to collect and measure biomarkers via urinary and blood serum. Each measure, urine or blood based, can be represented in multiple units of measure. For instance, creatinine is a biomarker that can be measured as milligrams per deciliter (mg/dl) or micromoles per liter (μmol/l). Here in the USA and in most of Europe, creatinine is most often reported in units of mg/dl. In other countries, such as Canada, Australia and some other European countries, units are displayed in μmol/l (14). Both units of measure for creatinine were measured by NHANES. In creating MetSRR, we reviewed the literature to identify the unit most clinically valid in the USA, when possible. Thus, for creatinine, the mg/dl measure was selected for use. However, there are examples when the less common unit of measure selected for inclusion when data were present and consistent across all cycles.

#### NHANES: data capture and importation into R

NHANES provided source code for transferring/importing data files into SAS. However, as discussed above, the open-source programming language, R, was selected for this project requiring that we create R-specific source code for each variable. Each variable file for each cycle was downloaded to a specified directory prior to importing into R. No authorization was required to access these public files. The following steps were executed in the creation of the MetSRR Explorer dataset.

- We first accessed NHANES’ publicly available data using the following link: https://www.cdc.gov/nchs/data_access/ftp_data.htm.
- Next, we selected the link on the landing page entitled, Data Sets and Related Documentation.
- Then we performed the variable search methods stated above to access data files and supplemental materials for each variable.
- Once a variable search was completed, we navigated to the component link Tables to access variable Data file names, Doc Files, Data Files and the publication date.
- We then accessed the doc file to view data file component descriptions including laboratory collection methods, units of measure, data processing information and analytic notes.
- Once the presence and units of each variable were verified in the Doc File, we selected and downloaded the data file that contained the selected variable.
- Once files for cycles 1999–2016 were downloaded, we used R coding language to import and manipulate the files.
- We used R’s open-source ‘foreign’ package to reformat each file because NHANES data files are not compatible with R.
- Once imported each data file was curated to extrapolate selected MetSRR variables and concatenated into master files by cycle years.
- Then we combined the master files into one MetS-specific working dataset.
- Following curation and cleaning of the dataset, the dataset was characterized and analyzed for web application.

#### Creating MetS indicator variables

Decision tree analysis, commonly used in biomedical informatics, can be employed as a rapid identification tool for classifying MetS (8). To interpret results of such analyses, an indicator variable must be created and included in datasets to discriminate between individuals displaying the phenotype (in this case, of MetS) from those not displaying the phenotype. Using the Adult Treatment Panel III (ATP III) diagnostic guidelines for MetS, we created nine classifying variables with binary designations that segregate individuals with MetS from those without MetS. These binary designations were then summated to create our single MetS indicator variable. According to the ATP III diagnostic criterion, a person is considered to have MetS if they meet any three of the following five criteria (note: sex-specific measures present):

- Triglyceride levels ≥150 mg/dL
- Blood Pressure ≥130/85 mm Hg
- Fasting Glucose ≥110 mg/dL
- Waist Circumference ≥102/≥88 (male/female)
- HDL < 40/< 50 (male/female)

Prior to summation, we created the following binary designations:

- Triglyceride levels ≥150 were given a code of 2, and participants below the threshold were designated as 1.
- Systolic blood pressure ≥130 were given a code of 2, and participants below the threshold were designated as 1.
- Diastolic blood pressure ≥85 were given a code of 2, and participants below the threshold were designated as 1.
- Glucose >110 were given a code of 2, and participants below the threshold were designated as 1.
- Male waist circumference ≥102 were given a code of 2, and participants below the threshold were designated as 1.
- Female waist circumference ≥88 were given a code of 2, and participants below the threshold were designated as 1.
- Male HDL <40 were given a code of 2, and participants below the threshold were designated as 1.
- Female HDL <50 were given a code of 2, and participants below the threshold were designated as 1.

Then we created a column to total the new binary variables. Lastly, a MetS indicator variable was created. All observations with a sum of 6 or greater were classified as positive for MetS and designated a code of 2. All observations that had a sum <6 were considered to be non-MetS positive individuals and were designated a code of 1.

#### Database design and web application implementation

The MetSRR online database and data visualization platform is implemented using the R packages Shiny and Data Tables (DT). Shiny is used to generate the user interface (UI) and process user input for custom data visualization and display of descriptive statistics. All GUI elements are created using Shiny, including the data Table, which is first rendered by DT then displayed through Shiny. Shiny also handles user interaction with the data Table and allows for retrieval of user-selected data for calculating descriptive statistics and data visualizations.

All calculations and plotting are performed using standard R functions. The MetSRR data is read in by R from a .csv file and rendered into a data Table using DT. DT executes the creation of the data Table, sets data Table properties concerning appearance and behavior and allows for advanced filtering and interactivity for data selection.

The MetSRR Explorer portion of the database is hosted on shinyapps.io as a Shiny app, which enables utilization of R-based packages on the live website. The Explorer application hosted on shinyapps.io is then embedded into the WordPress-based healthdisparityinformatics.com/MetSRR site using the WordPress plugin iframe, which allows for embedding separate HTML pages into WordPress pages by entering the MetSRR shiny app’s URL into short code with iframe tags. This provides easy access to the MetSRR database through the parent ‘healthdisparityinformatics’ site.

## Results and discussion

**Figure 3.**
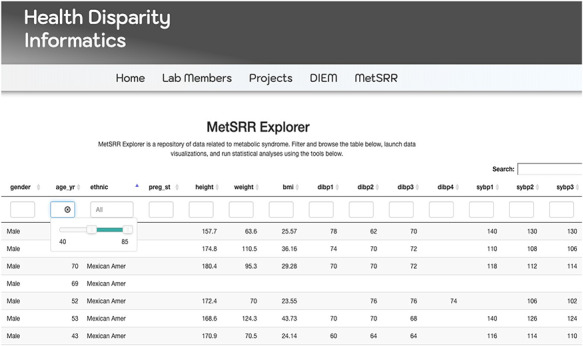
Image only reflects a fraction of the MetSRR Explorer. Observations too numerous to capture in a single image.

**Figure 4.**
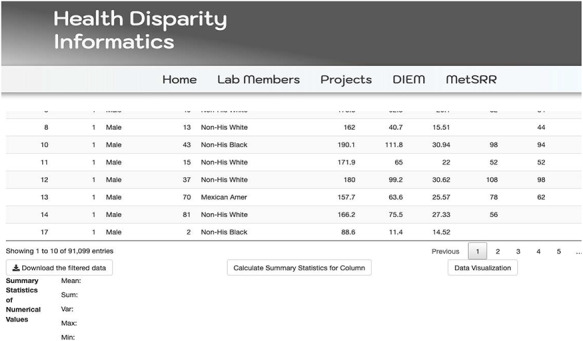
Illustration of MetSRR Explorer features including download capability.

The MetSRR interactive database has features that enable the user to curate datasets specific to their research interests. Figure 3 below shows the standard interface one would see when accessing the MetSRR Explorer. Users have the ability to customize datasets by simply clicking the empty box below the variable name they are looking to parse. This is illustrated for the age_yr variable in Figure 3, where the age range can be customized by using the bar to adjust it. Figure 4 shows the area under the Table that gives the user the option to download a .csv file once they select the button ‘Download the filtered dataset’. Once downloaded the file can be opened and analyzed in software such as excel, R, python and many others.

**Figure 5.**
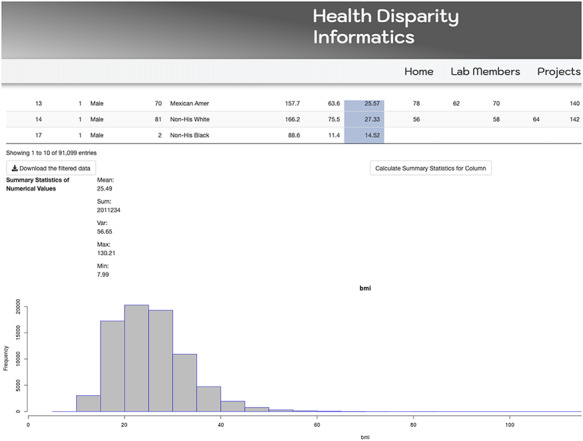
MetSRR visualization of the distribution of the BMI variable.

Our database has data visualization capacities, as well. The Tables provide a wide array of information including descriptive statistics, characterization of variables and scholastic references validating the inclusion of selected novel biomarkers associative of MetS. Descriptive statistics can be visualized via MetSRR Tables or in the explorer. Figure 5 depicts descriptive statistics and data visualization for a selected numerical variable in our MetSRR Explorer. Table 1 (All Tables located at: http://healthdisparityinformatics.com/Tables.) is an illustration of our datasets descriptive statistics centralized in one location.

**Table 1.** MetSRR descriptive statistics

Variable	Mean	SD	Median
Age (yr)	31.28	± 24.77	24
Height (cm)	156.7	± 22.71	162.4
Weight (kg)	62.3	± 31.19	65.2
BMI (kg/m^2^)	25.49	± 7.53	24.71
Diastolic BP 1 (mmHg)	66.24	± 14.76	66
Diastolic BP 2 (mmHg)	66.11	± 14.86	68

MetSRR’s curated descriptive statistics including mean, median and standard deviation provides additional statistical support. This coupled with frequency and data visualization of categorical variables guides users in appropriate statistical modeling. With the ability to visualize mean, median and histograms for numeric variables, MetSRR allows the user to make informed decisions regarding pre-experimental analyses, for instance, identifying the suitable parametric tests for variables. In the case that variable data are skewed, MetSRR has the capability to look at the histogram and evaluate the standard deviation then employ a more suitable non-parametric test such as Spearman correlation. The benefits of MetSRR can save time for researchers who are familiar and unfamiliar with computation research.

MetSRR Tables provide a new level of support by providing statistical variable classification. These tables identify each variable as a continuous, discrete or categorical variable. MetSRR also identifies variable data types presented in R, distinguishing these variables as an integer, factor or numeric variable. MetSRR translates statistical variable data types to programming variable data types. MetSRR also streamlines the process of variable manipulation and increases understanding of specific applicable mathematical models.

**Figure 6.**
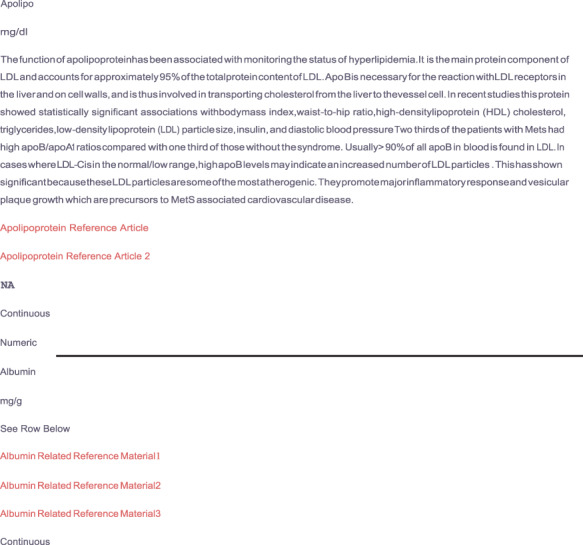
Depicts supplemental material provided by the MetSRR database. The live links allow users to access journal articles supporting the inclusion of selected variables.

Other tables in our database provide insight to the current literature around MetS. The scope of the provided scholastic materials include current methods of MetS biomarker discovery, current clinically evaluative biomarkers of MetS, as well as research regarding the validity of inclusion of the novel markers selected. Figure 6 depicts a portion of the variable characterization and reference table. Illustrated in this figure are the descriptions of the apolipoprotein and albumin variables. Here we provide variable names, the unit of measure used in the dataset, an explanation of inclusion, scholastic reference material, statistical variable type and R variable type. The read text indicates the live link that takes the user directly to the cited article referenced for that variable.

## Conclusion

The power of MetSRR lies in the data it has captured. By including the range of biometric data to surveyed demographic and ancillary data, MetSRR reflects the many and complex aspects of MetS, particularly for interrogation regarding health disparities among racial, ethnic, biological gender, socioeconomically striated or other subsets of individuals. The descriptiveness of the available datasets and curated supplemental materials enhances the utility of MetSRR. An individual utilizing MetSRR will have, at-a-glance, data concerning a range of variables that will better articulate experimental questions, as well as the types of statistical methods that can be used in evaluation.

In other areas of informatics, such as bioinformatics, there are repositories such as TCGA that solely house ‘omics’ data (1). Vetting useful clinical data requires the presence of current and historical clinical interventions, notation of environmental and socioeconomic history and clinical measurements that span multiple modalities. Due to this complexity, biocuration of clinical data consistently lags behind data generation in funding, development and recognition (15). MetSRR provides a framework for closing the gap by increasing accessibility and understanding the petabytes of clinical data that has been amassed. When data are curated to a high standard and made accessible, the ability of researchers to cognize already available information is increased. As a result, researchers can generate meaningful hypotheses dedicated to further the comprehension of MetS etiology and the subsequent health disparity of MetS. Efficient hypothesis generation allows specific pre-experimental analysis leading to greater experimental accuracy as it relates to the human condition, specificity and understanding.

Feedback from users of MetSRR will inform further enhancements to MetSRR for MetS focused researchers. As the Tables and datasets housed at healthdisparityinformatics.com/MetSRR continue to be updated with newly acquired data, we anticipate that MetSRR will grow in parallel by querying across various databases in the same manner that TCGA does for cancer-based studies. Even in its present state, MetSRR provides the opportunity for improvements in patient-specific diagnosis, prevention and treatment methods.
